# Mental health and COVID-19 pandemics: The worrisome humanitarian perspective from the Middle East

**DOI:** 10.7189/jogh.11.03014

**Published:** 2021-01-30

**Authors:** Dina Sabry Said, Gianluigi Lopes, Liliana Lorettu, Gabriele Farina, Catello M Panu Napodano, Antonella Amadori, Giuseppe Pichierri, Luca Cegolon, Susanna Padrini, Saverio Bellizzi, Yehia Alzoubi

**Affiliations:** 1 College of Business Administration, American University of the Middle East, Kuwait; 2 Public Health Specialist, Independent Consultant, Geneva, Switzerland; 3 University of Sassari, Sassari, Italy; 4 Kingston Hospital NHS Foundation Trust, Microbiology Unit, Kingston Upon Thames, UK; 5 Local Health Unit N.2 “Marca Trevigiana”, Public Health Department, Treviso, Italy; 6 AISPO Associazione Italiana per la Solidarieta’ tra i popoli, Milan, Italy; 7 Medical Epidemiologist, Independent Consultant, Geneva, Switzerland

A recent survey conducted by the World Health Organization (WHO) from June to August 2020 around the globe has clearly highlighted how the COVID-19 pandemic has disrupted or halted critical mental health services in 93% of the 130 countries under study despite the demand for mental health is increasing [1].

Specifically, 45 countries reported disruption of emergency interventions such as those for people experiencing severe substance use withdrawals syndromes. On the other hand, over 60% of countries reported disruption for vulnerable people, including children and adolescents (72%), the elderly (70%), and women requiring antenatal or postnatal services (61%). Also, around three out of four countries under study reported at least partial disruption of school and workplace mental health services [1].

The WHO Eastern Mediterranean Region, which comprises 21 Member States (Afghanistan, Bahrain, Djibouti, Egypt, Iran, Iraq, Jordan, Kuwait, Lebanon, Libya, Morocco, Oman, Pakistan, Qatar, Saudi Arabia, Somalia, Sudan, Syria, Tunisia, United Arab Emirates and Yemen) and Palestine (West Bank and Gaza Strip), with a population of around 680 million people, hosts some of the most important protracted crises on earth that resulted in major population movement due to forced displacement and migration.

Conflicts in the Eastern Mediterranean Region are severely impacting human well-being, including mental health of displaced populations and host communities. One peculiar feature is represented by the fact that countries neighbouring those in conflict are under stress because of the large numbers of people seeking shelter. At the very beginning of 2019, 32.1 million out of the globally 70.8 million displaced persons originated from the Middle East area and 25.4 million continued to reside in the region [2].

**Figure:**
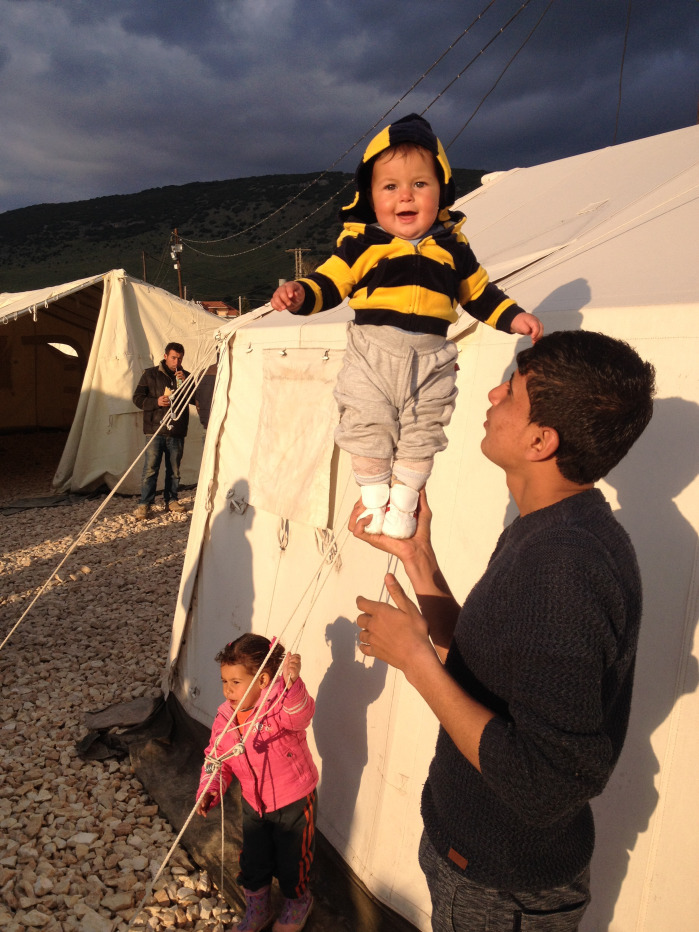
Photo: This photo, taken in a Syrian Refugee Camp in the North of Greece, shows the precarious situations of thousand people fleeing their own countries with their family, including several children (from the author’s own collection, used with permission).

Isolation, loss of income and fear due to the COVID-19 pandemic are exacerbating existing mental health conditions: common and severe mental disorders are more prevalent in humanitarian crises, especially among women and children, and those exposed to violence and forcible displacement. Specifically, estimates suggest that more than one in five people in post-conflict settings have depression, anxiety disorder, posttraumatic stress disorder, bipolar disorder, or schizophrenia [2].

To note how the current context may also worsen stigmatization of displaced people, often accused of bringing crime and disease, leaving them more vulnerable to mental health and psycho-social problems.

To give some examples, in Lebanon, multiple incidents of suicide were reported among refugees in April and there was a spike in instances of threats to self-harm and harm to others amidst domestic violence and family disputes. On the other hand, the Multi-sectoral Rapid Needs Assessment jointly conducted by WFP, UNICEF and UNHCR in Jordan in the same time-period showed that 41% of all respondents witnessed a negative impact on their children’s well-being because of the COVID-19 crisis. Finally, it is important to underline the devastating compounded effect of the current crisis on contexts like Yemen and Libya with their weak national health systems and the shortage of qualified mental health staff; the Libyan context in particular deserves close observation and action due to additional distress aspects like detention centres [3].

While more than 80% of high-income countries reported deploying telemedicine and teletherapy to bridge gaps in mental health, this proportion becomes far lower (50%) in low-income countries and is likely to be even lower in setting experiencing protracted conflicts [1].

As emphasized by the WHO, monitoring changes and disruption of services and prompt addressing of these issues should be an integral part of response and recovery plans along with the other components related to maintaining essential health services. The financial aspects of such as integration must be carefully taken into consideration: despite the fact 89% countries reported that mental health was part of their COVID-19 response plans, only 17% revealed full additional funding for covering these activities [1].

As framed by the Inter-Agency Standing Committee (IASC), establishing a mental health and psycho-social strategy is critical and should be inclusive of targeted actions towards COVID-19 cases, survivors, contacts, family members, frontline workers and the broader community, with special attention to the needs of vulnerable groups [4]. Fear, stigma, negative coping strategies and other needs should be prioritized to ensure a whole-of-society approach in fragmented and multivariate contexts of the Middle East.

The need to support mental health assistance for vulnerable populations can include a series of innovative interventions like telemedicine, which could be directly financed by the richer countries via recruiting the mental health specialists from the same language group and from around the world to try to give support and follow ups. Other possible solutions include the coordinated connection with facilities and clinics that have better and more specialized personnel. Indeed, a political solution at a higher level for stabilizing and developing the entire region is the most important factor.
